# Supplementary material for: Precision Oncology Through Dialogue: AI-HOPE-RTK-RAS Integrates Clinical and Genomic Insights into RTK-RAS Alterations in Colorectal Cancer
Source: Biomedicines. 2025 Jul 28;13(8):1835. doi: 10.3390/biomedicines13081835 (PMC12383457; doi:10.3390/biomedicines13081835)
Supplement: Supplementary file 1 [file biomedicines-13-01835-s001.zip › biomedicines-3716491-supplementary.pdf]

Supplementary Materials:

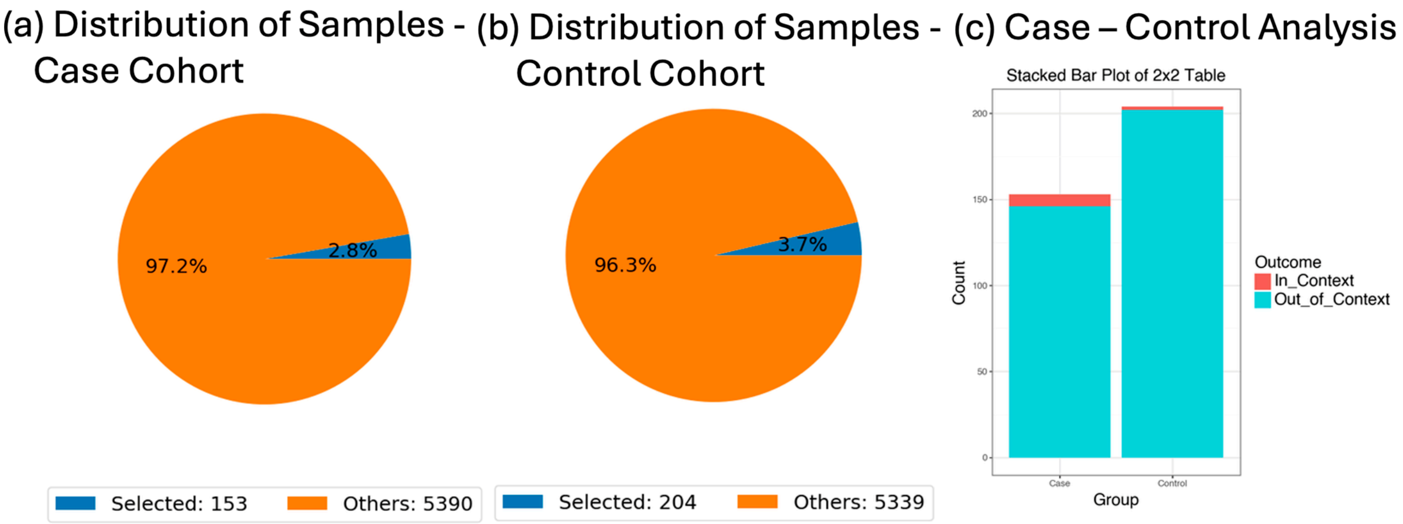

Figure S1. AI-HOPE-RTK-RAS analysis of CBL mutation frequency in early-onset versus late-onset colorectal cancer (CRC) patients. This figure illustrates AI-HOPE-RTK-RAS's capacity to evaluate gene-specific alterations, in this case focusing on CBL mutations, within demographically defined CRC cohorts. The platform was used to compare the prevalence of CBL mutations in early-onset CRC (EOCRC) versus late-onset CRC (LOCRC) Hispanic/Latino (H/L) patients using a natural language-driven odds ratio framework. a) The case cohort includes 153 EOCRC H/L patients under the age of 50 (2.8% of the dataset) identified using age and ethnicity filters. A pie chart visualizes the proportion of selected EOCRC cases relative to the full dataset. b) The control cohort consists of 204 H/L patients over the age of 50 with LOCRC (3.7% of the dataset). The pie chart shows their representation within the population. c) An odds ratio test compares the presence of CBL mutations across both groups using a 2x2 contingency table and stacked bar plot. CBL mutations were observed in 4.58% of EOCRC and 0.98% of LOCRC samples. The resulting odds ratio was 4.842 (95% CI: 0.992–23.647, p = 0.071), indicating a nearly fivefold increase in CBL mutation odds in early-onset cases, though the result did not reach conventional statistical significance. This trend highlights a potential enrichment of CBL mutations in EOCRC H/L patients and demonstrates AI-HOPE-RTK-RAS's utility for uncovering subgroup-specific genomic patterns that may warrant further investigation in larger cohorts.

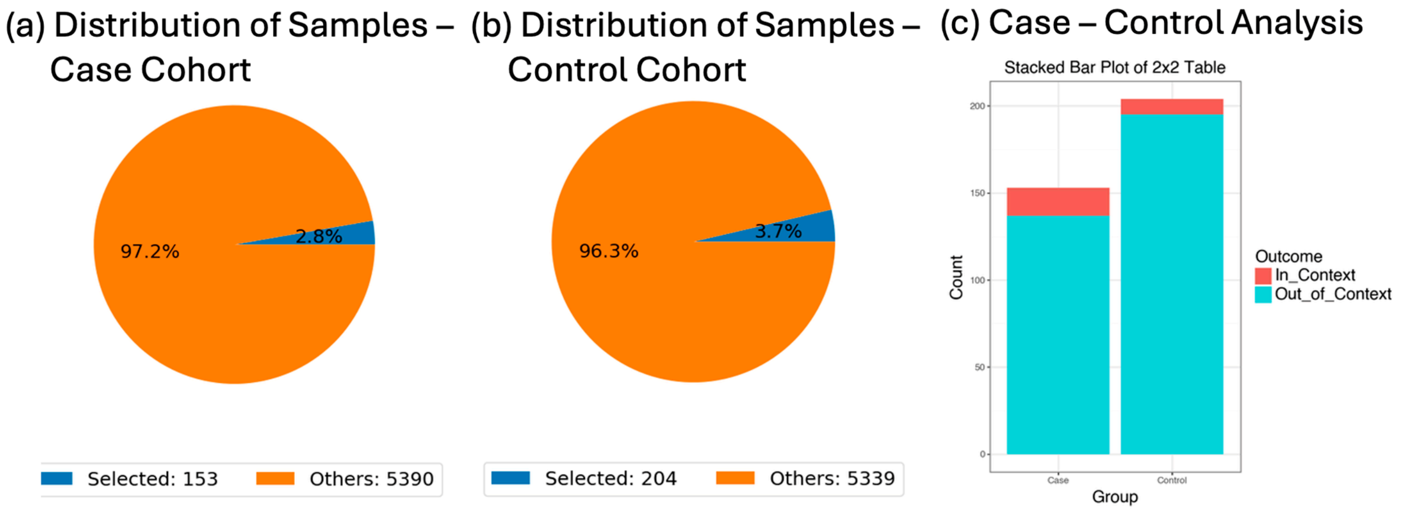

Figure S2. AI-HOPE-RTK-RAS evaluation of NF1 mutation frequency in early-onset versus late-onset colorectal cancer (CRC) patients. This figure demonstrates AI-HOPE-RTK-RAS's functionality

in detecting age-related genomic differences through natural language-driven analysis, focusing on *NF1* mutation prevalence among Hispanic/Latinos (H/L) CRC patients. a) The case cohort includes 153 EOCRC H/L patients under the age of 50 (2.8% of the total dataset), identified using filters for both age and ethnicity. A pie chart illustrates the proportion of selected EOCRC H/L patients relative to the overall CRC cohort. b) The control cohort consists of 204 LOCRC H/L patients over the age of 50 (3.7% of the dataset) selected using the same ethnicity criteria. A pie chart shows this group's relative representation. c) An odds ratio test was used to compare the frequency of *NF1* mutations between the EOCRC and LOCRC groups. The bar plot displays a 2x2 comparison of *NF1*-mutated (in-context) versus wild-type (out-of-context) samples. *NF1* mutations were observed in 10.46% of EOCRC samples and 4.41% of LOCRC samples. The odds ratio was 2.53 (95% CI: [1.087, 5.893], $p = 0.045$), indicating that early-onset patients had more than twice the odds of harboring *NF1* mutations compared to their later-onset counterparts. This statistically significant difference supports a potential role for *NF1*alterations in the molecular landscape of early-onset CRC among H/L populations and highlights the utility of AI-HOPE-RTK-RAS for uncovering clinically relevant, subgroup-specific genomic patterns.

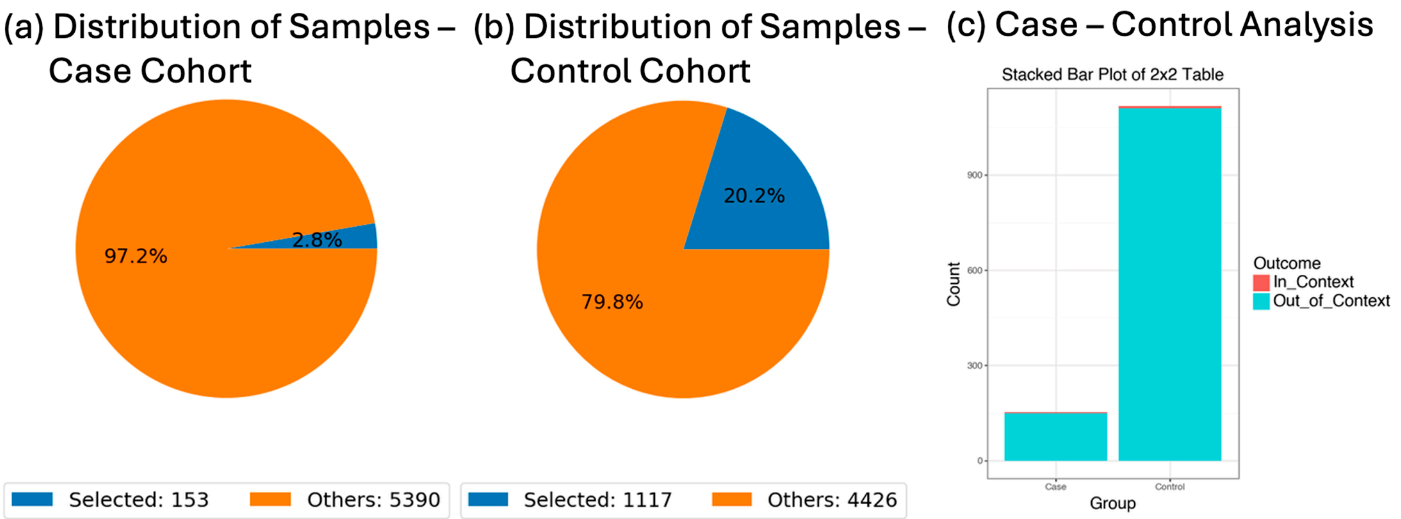

Figure S3. AI-HOPE-RTK-RAS analysis of MAPK3 mutation frequency in early-onset colorectal cancer (CRC) patients by ethnicity. This figure illustrates AI-HOPE-RTK-RAS's ability to identify ethnicity-associated genomic alterations through natural language-driven querying, specifically focusing on *MAPK3* mutations in early-onset colorectal cancer (EOCRC) patients. a) The case cohort includes 153 EOCRC Hispanic/Latino (H/L) patients under the age of 50 (2.8% of the dataset) selected using demographic filters based on ethnicity. A pie chart displays the proportion of EOCRC HL patients among all samples. b) The control cohort consists of 1,117 EOCRC non-Hispanic White (NHW) patients under age 50 (20.2% of the dataset), identified based on combined race and ethnicity filters. A corresponding pie chart visualizes this group's relative dataset representation. c) An odds ratio test compares the frequency of *MAPK3* mutations between the two cohorts. As shown in the stacked bar plot, *MAPK3* mutations were detected in 2.61% of EOCRC HL samples and 0.63% of EOCRC NHW samples. The odds ratio was 4.26 (95% CI: [1.232, 14.715], $p = 0.043$), indicating that H/L EOCRC patients had more than four times the odds of harboring a *MAPK3* mutation compared to their NHW counterparts. This statistically significant finding highlights a potential enrichment of *MAPK3* mutations in the Hispanic/Latino early-onset CRC population and supports the need for further investigation into *MAPK3* as a possible biomarker or driver of ethnicity-specific molecular differences in EOCRC.

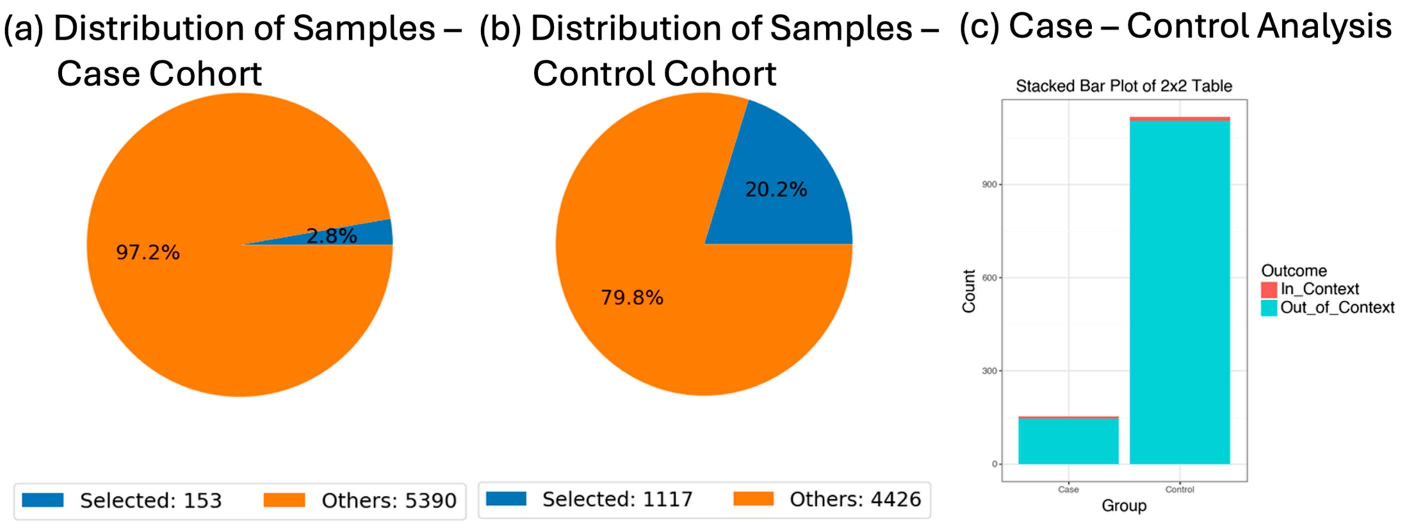

Figure S4. AI-HOPE-RTK-RAS analysis of CBL mutation frequency in early-onset colorectal cancer (EOCRC) patients by ethnicity. This figure demonstrates AI-HOPE-RTK-RAS's capability to identify ancestry-related mutation patterns through natural language-driven analysis, focusing on CBL mutations in EOCRC patients. a) The case cohort consists of 153 Hispanic/Latino (H/L) EOCRC patients under the age of 50 (2.8% of the dataset) selected using ethnicity-specific filters. A pie chart illustrates the proportion of selected cases within the total CRC dataset. b) The control cohort includes 1,117 non-Hispanic White (NHW) EOCRC patients under age 50 (20.2% of the dataset), identified through combined race and ethnicity filters. A corresponding pie chart displays their relative representation. c) An odds ratio test compares the frequency of CBL mutations between the two groups. As visualized in the stacked bar plot, CBL mutations were present in 4.58% of EOCRC H/L cases and 1.16% of EOCRC NHW cases. The calculated odds ratio was 4.07 (95% CI: [1.599, 10.37], p = 0.005), indicating that H/L EOCRC patients had more than four times the odds of harboring a CBL mutation compared to their NHW counterparts. This statistically significant enrichment suggests that CBL may play a more prominent role in the molecular landscape of early-onset CRC in H/L patients and supports its potential relevance as a biomarker or oncogenic driver in this population.

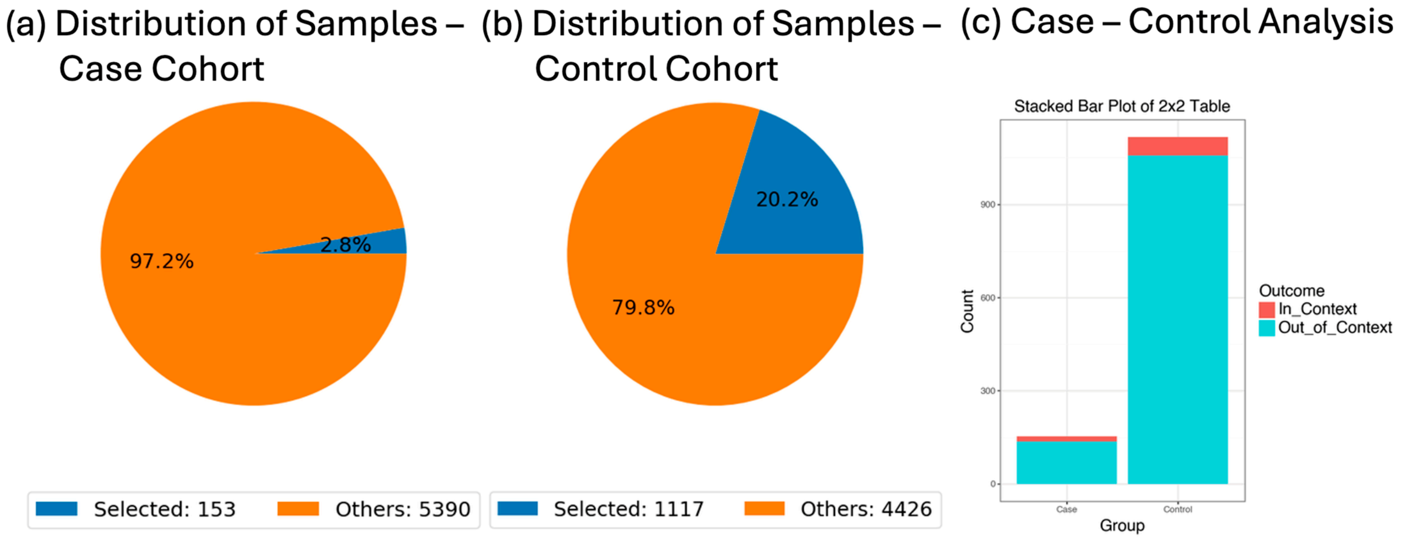

Figure S5. AI-HOPE-RTK-RAS analysis of NF1 mutation frequency in early-onset colorectal cancer (EOCRC) patients by ethnicity. This figure demonstrates AI-HOPE-RTK-RAS's capability to uncover ancestry-associated genomic alterations using natural language-driven queries, focusing on *NF1* mutation prevalence in early-onset colorectal cancer (EOCRC). a) The case cohort includes 153 Hispanic/Latino (H/L) EOCRC patients under the age of 50 (2.8% of the dataset) selected based

on ethnicity filters. A pie chart illustrates the proportion of selected H/L patients within the overall cohort. b) The control cohort consists of 1,117 non-Hispanic White (NHW) EOCRC patients under the age of 50 (20.2% of the dataset), identified through race and ethnicity filtering. The pie chart reflects the representation of this group in the dataset. c) An odds ratio test evaluates the frequency of *NF1* mutations between the two cohorts. The stacked bar plot visualizes the comparison of in-context (*NF1*-mutated) versus out-of-context samples in both groups. *NF1* mutations were found in 10.46% of EOCRC HL samples and 5.37% of EOCRC NHW samples. The odds ratio was 2.06 (95% CI: [1.153, 3.673], p= 0.021), indicating that Hispanic/Latino patients had approximately double the odds of harboring an *NF1* mutation compared to NHW patients. This statistically significant enrichment suggests that *NF1* may play a more prominent role in the tumor biology of early-onset CRC in Hispanic/Latino populations and highlights AI-HOPE-RTK-RAS's utility in conducting ancestry-aware, gene-specific analyses to inform precision oncology.

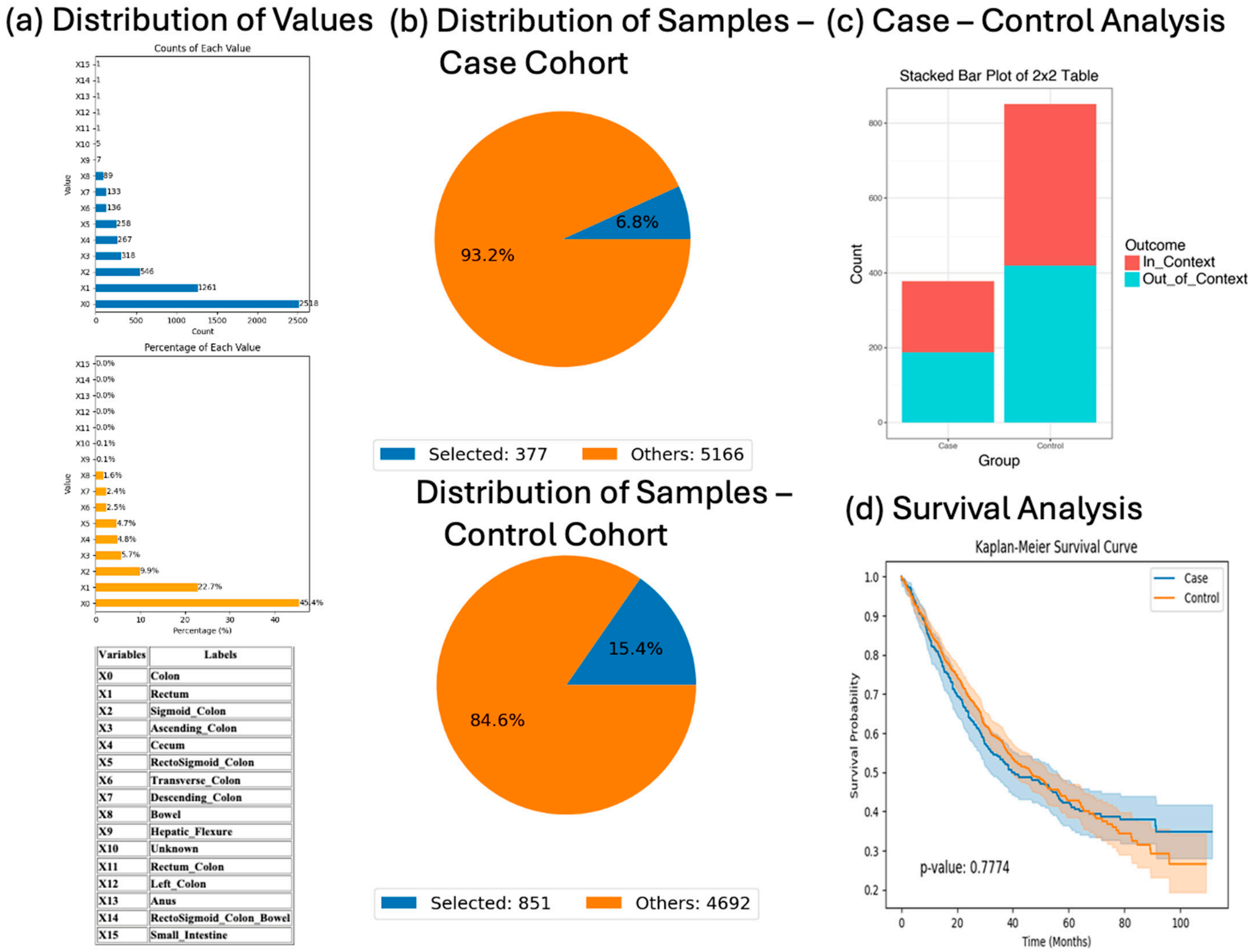

Figure S6. AI-HOPE-RTK-RAS analysis of colorectal cancer (CRC) tumors with KRAS mutations by tumor location: proximal vs. distal colon. This figure demonstrates the use of AI-HOPE-RTK-RAS to compare clinical outcomes and demographic patterns in KRAS-mutated CRC patients based on tumor location—proximal (case group) versus distal (control group)—with an additional odds ratio analysis for sex-based enrichment. a) The case and control groups are defined by the primary tumor site. The bar charts show the frequency and proportion of CRC primary sites across the dataset. Proximal sites (e.g., Cecum, Ascending Colon, Transverse Colon) are grouped as the case cohort, while distal sites (e.g., Sigmoid Colon, Rectum) form the control. The Colon (X0), Sigmoid Colon (X2), and Ascending Colon (X3) are among the most frequently represented locations, with the majority of tumors arising in the distal colon. b) Pie charts illustrate the number of selected

samples from each cohort relative to the dataset. The proximal (case) cohort includes 377 KRAS-mutant samples (6.8%), while the distal (control) cohort includes 851 samples (15.4%). This visualization highlights the relatively lower frequency of proximal tumors with KRAS mutations in the analyzed dataset. c) An odds ratio test evaluates sex-based enrichment by comparing the proportion of female patients between cohorts. The stacked bar chart shows the number of in-context (female) and out-of-context (non-female) samples for both groups. The odds ratio was 0.985 (95% CI: [0.773, 1.256], $p = 0.955$), indicating no significant difference in female representation between the proximal and distal KRAS-mutant tumor sites. d) Kaplan–Meier survival curves compare overall survival between proximal and distal CRC patients with KRAS mutations. While the curves appear similar and intersect at later timepoints, the survival difference is not statistically significant ($p = 0.7774$), suggesting comparable outcomes across tumor locations in this KRAS-mutant subgroup. Confidence intervals are shown for both groups, confirming the robustness of this observation.

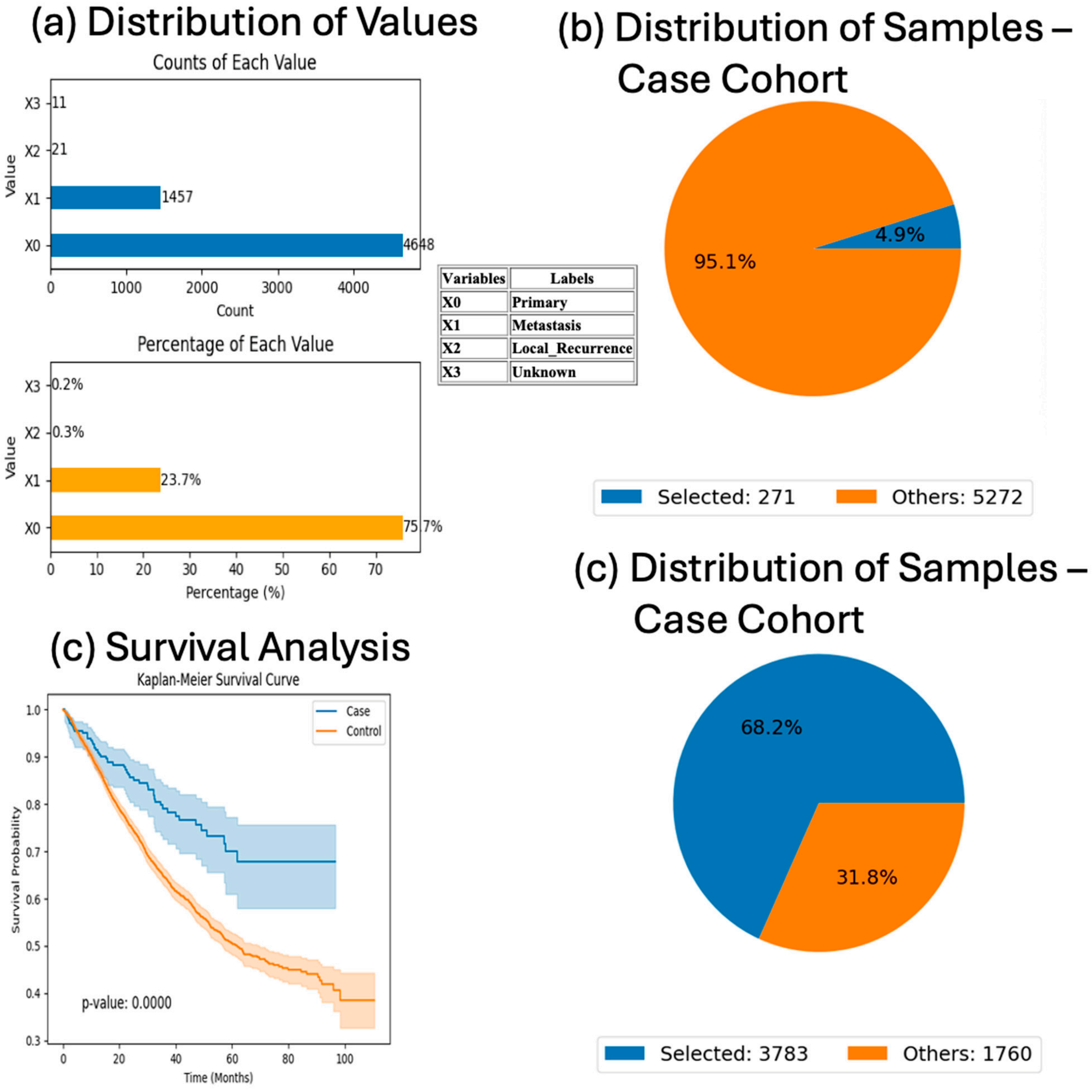

Figure S7. AI-HOPE-RTK-RAS analysis of primary colorectal cancer (CRC) tumors with and without NF1 mutations. This figure showcases AI-HOPE-RTK-RAS's ability to explore clinical outcomes in colorectal cancer (CRC) patients by comparing primary tumors with and without *NF1* mutations. a) The analysis begins by filtering for primary tumor samples within the CRC dataset. The bar charts summarize the distribution of tumor sample types. Primary tumors (X0) represent the

majority (n = 4,648; 75.7%), followed by metastases (X1; 23.7%) and rare classifications such as local recurrence and unknown. This selection ensures that the subsequent comparison focuses solely on primary CRC. b) Two cohorts are generated based on *NF1* mutation status. The case cohort includes 271 primary CRC tumors with *NF1*mutations (4.9%), while the control cohort includes 3,783 primary tumors without *NF1* mutations (68.2%). Pie charts visually represent the selected subsets relative to the entire dataset, illustrating the difference in mutation frequency across the primary tumor landscape. c) Kaplan–Meier survival curves compare overall survival between the *NF1*-mutated and wild-type groups. The survival difference is statistically significant ($p = 1 \times 10^{-5}$), with the *NF1*-mutated cohort showing a markedly improved survival probability over time. Confidence intervals are shaded to reflect statistical robustness. These results suggest that *NF1* mutations may confer a favorable prognostic impact in primary CRC and highlight the utility of AI-HOPE-RTK-RAS in facilitating rapid, mutation-specific survival analyses across clinically relevant subgroups.
